# Dr. Henry Patterson Loomis

**Published:** 1908-01

**Authors:** 


Dr. Henry Patterson Loomis, of New York, professor of therapeutics and clinical medicine at Cornell University, died from pneumonia at his home December 22, 1907, aged 48 years. He was a son of the late Dr. Alfred L. Loomis and a former president of the American Academy of Medicine. He is survived by Mrs. Loomis and two children.

